# Combating antiscience: Are we preparing for the 2020s?

**DOI:** 10.1371/journal.pbio.3000683

**Published:** 2020-03-27

**Authors:** Peter J. Hotez

**Affiliations:** 1 Texas Children’s Hospital Center for Vaccine Development, National School of Tropical Medicine, Baylor College of Medicine, Houston, Texas, United States of America; 2 Department of Biology, Baylor University, Waco, Texas, United States of America; 3 Hagler Institute for Advanced Studies at Texas A&M University, Texas, United States of America; 4 Scowcroft Institute of International Studies, Bush School of Government and Public Service, Texas A&M University, Texas, United States of America; 5 James A Baker III Institute of Public Policy, Rice University, Houston, Texas, United States of America

## Abstract

In the last half of the 2010s, we saw an upswing in antiscience movements and unprecedented attacks on scientists in the United States and elsewhere. All indications suggest that this trend will not slow or reverse anytime soon, and it is now increasingly apparent that it will fall to the scientists themselves to respond, engage a skeptical public, and lead the defense of science. Accordingly, we must recognize opportunities to both reorganize science doctoral and postdoctoral training and incentivize senior scientists as a means to establish a new ecosystem for science public engagement. Such activities may become essential if the assaults on our profession continue or expand. Today, the commitment of young scientists to public service is at an all-time high. However, we must work quickly to capture that enthusiasm and channel it into a social good, lest we lose this opportunity. Potentially, open-access publishers could play a central role.

## Abstract 2

This Perspective article discusses the problem that as antiscience movements grow and gain ascendancy, it may increasingly fall to the scientists to mount a defense or lead a response; however, many universities discourage scientists from speaking out, while fewer provide public engagement training at predoctoral and postdoctoral levels.

The last decade was remarkable for a rise in antiscience activities, especially in the areas of climate change, air pollution, and synthetic chemicals, to name a few. In the biological and biomedical sciences, vaccines and vaccination programs became targets, as did gene editing, genetically modified crops, and other biotechnologies. Antiscience activists linked illnesses such as cancer or Lyme disease to conspiracies and cover-ups. In parallel, several populist governments worked to enact restrictions or slash budgets to imperil the future of research institutions in Brazil, Hungary, Italy, and the United States [1–3]. Some have voiced concerns that we might enter a new “post-truth” era [4], which could mean an expansion of attacks on both science and scientists.

One of the most prominent and visible antiscience movements is active in North America and Europe and focused on discrediting vaccines. As both a vaccine scientist and autism advocate for my adult daughter Rachel, I have been targeted by antivaccine activists for more than 2 decades. However, these harassments accelerated around the time leading up to the 2016 presidential election campaign and then reached new levels in 2019 as public health deteriorated due to declines in vaccinations [5–7]. Here, I give a first-hand account of my experiences (as a type of case study) and use them to propose potential reforms in graduate and postdoctoral science training as a mechanism for countering antiscience.

The year 2019 was a bad one in terms of the return of vaccine-preventable diseases to the US and Europe. Measles cases in Europe exceeded 100,000 and surpassed 1,000 in the US because of sharp declines in immunizations with the measles-mumps-rubella (MMR) vaccine, while thousands of teenagers were denied cancer prevention through the human papillomavirus (HPV) vaccinations, and we saw many pediatric flu deaths among unvaccinated children despite recommendations [8–10]. The decline in vaccinations is the result of several factors, but in the US and many European nations, it is strongly linked to a strengthened, empowered, and emboldened antivaccine movement [9, 11–13]. Whereas in the past the antivaccine movement was mostly a fringe element, it gained strength and critical mass to a point where it has affected public health. It now dominates the internet with hundreds of misinformation websites, amplified on social media and with political links to populist movements [14]. Their major assertions are as follows: (1) vaccines can cause autism or chronic illnesses; (2) these conditions represent a form of “vaccine injury”; (3) vaccines are unregulated and not adequately tested for safety; (4) vaccines contain toxic ingredients; (5) parents must assert their “health freedom”; and/or (6) there is a conspiracy between the Centers for Disease Control and Prevention and vaccine manufacturers to “push” vaccines on an unsuspecting public. In 2019, the World Health Organization listed “vaccine hesitancy” as a leading global health threat [15].

Too often, the public health community has been slow or halting in its response to the antivaccine movement. In the US, for example, the US Congress held hearings on the antivaccine movement in February and March of 2019, but they produced few, if any, tangible outcomes. In the meantime, the media and political activities of the antivaccine movement continue to expand and run mostly unopposed.

## Defending science

As a pediatrician scientist who develops vaccines for poverty-related neglected diseases, and as the parent of an adult daughter with autism, I felt uniquely positioned to both defend vaccines and counter the fake messaging coming from the antivaccine lobby. In 2018, I wrote a book, entitled *Vaccines Did Not Cause Rachel’s Autism*: *My Journey as a Vaccine Scientist*, *Pediatrician*, *and Autism Dad* (Johns Hopkins University Press), to fill a void in the literature–explaining the genetic underpinnings of autism as well as the data refuting its links to vaccines. I felt that adding a personal story to the public dialogue on this issue might ultimately bridge a gap for both the community of scientists and the families dealing with the realities of the autism spectrum [16].

I hoped that by explaining the science of autism—how it begins in early fetal brain development through the actions of dozens if not hundreds of autism genes and epigenetic mechanisms—it would help make the evidence showing that vaccines did not cause autism easier to grasp. For example, through whole-exome sequencing on Rachel, my wife Ann, and myself, we identified a potentially new autism gene possibly belonging to a larger group of recently published autism genes encoding neuronal cytoskeleton proteins [17].

I also felt it was important to put a face on science and scientists. A study conducted by Research!America finds that almost no US citizens can name a living scientist, and the few who did would name individuals such as Bill Nye and Neil deGrasse Tyson. I’m a fan of both Nye and Tyson, but for me, the survey essentially said that the typical working scientist–who struggles daily with complicated lab meetings and experiments, revising scientific papers, resubmitting grant applications, and presenting at scientific meetings in front of critical audiences—is mostly invisible. The US public has little knowledge about what scientists actually do.

Although *Vaccines* was not my first book, it was certainly the most personal, exposing my family and myself to public scrutiny. I felt this was important to do in order to be effective at communicating to worried and vaccine-hesitant parents. So far, the book has been relatively well received by the scientific community and several academic societies, and I have gotten many heartfelt emails and letters from parents thanking me for my candor, scientific explanations, and willingness to speak out and defend vaccines. Beyond the book, I have also since written several op-eds and given both public lectures on the topic (including at annual meetings of academic societies and at universities) and done interviews and podcasts. It is difficult to measure the impact of my vaccine advocacy, but based on correspondence and interactions with parents and healthcare professionals across the country, I get the sense that for many, my public remarks are often the first time they are hearing a positive message about vaccines and their first interaction with an actual scientist.

There was, however, a downside of this public engagement: intense exposure to an antivaccine media machine. It pelts my Amazon.com site with one-star reviews or makes outrageous statements on social media. What has been more worrisome has been the stalking or protests at meetings where I speak and some specific postings by leaders of the antivaccine movement. In December of 2019, Robert F. Kennedy Jr. posted to his Instagram account a statement claiming that I am an “industry shill” for the multinational vaccine companies [18]. His evidence is that I receive grant funding from the Gates Foundation and other sources, including a start-up biotech in the 1990s, perhaps not understanding that principal investigators require grant funds to support research scientists, experiments, and reagents. He also digressed about Jonas Salk, saying that he is one of my scientific heroes, which is true, but then went on to falsely accuse Dr. Salk of nefarious activities. My worry is that one of Mr. Kennedy’s many followers might believe his posting and try to escalate their attacks.

## A larger response to antiscience

The antivaccine movement is highly visible, but it is not the only antiscience movement gaining in strength and access. I loosely define an “antiscience movement” as an organized and funded rejection of science and scientific principles and methods in factor of alternative views, often linked to the targeting or harassment of individual scientists. We are now seeing this play out in other areas, including those highlighted earlier. I am especially concerned that biomedicine’s latest and cutting-edge biotechnologies such as OMICs and CRISPR gene editing will come under attack and block scientific progress that require these approaches. Without substantial efforts to counter them, I believe that organized antiscience movements will continue to gain ascendancy in the coming decade of the 2020s.

There has been some progress. In 2018, the American Academy of Arts and Sciences issued an important report on *The Perceptions of Science in America*, which is part of a larger *Public Face of Science Initiative* [19]. Its major findings include the somewhat reassuring message that public confidence in science and scientific investments have remained stable over the last 3 decades, although levels of confidence can vary significantly depending on educational attainment, race, age, and political ideologies; also, there is a need to understand why science skepticism is particularly strong among certain groups [19]. Moreover, the American Association for the Advancement of Science (AAAS) and the National Academies of Science, Engineering, and Medicine (NASEM) both feature important discussions and workshops on the rise of antiscience, particularly as it pertains to agencies of the US federal government, while the former hosts the important Center for Public Engagement with Science and Technology. Both the Union of Concerned Scientists and the Committee of Concerned Scientists have worked to defend us from attacks. Prominent science historians and philosophers have also addressed the rise of organized antiscience [20], and there is an entire journal devoted to the public understanding of science [21].

Ultimately, combating antiscience movements and their organized activities will require a complex response and, likely, the involvement of major governments working in public–private partnerships. For the biomedical sciences, and possibly other fields, I believe that a critical element must include expanded visibility for scientists themselves, who are both “in the mix”—meaning active in their fields, publishing papers, writing grants, and speaking at scientific meetings—and simultaneously comfortable with engaging the public and who are versatile in using modern tools of written and oral communication.

## Training a new generation of “visible scientists” and science communicators

There are now some excellent programs of science communication emphasizing science journalism. They include master’s programs at Boston University, Columbia, Johns Hopkins, Massachusetts Institute of Technology, New York University, University of California, Santa Cruz, and Texas A&M University, as well as Knight Science Journalism Fellowships [22]. Imperial College London has also built out an excellent and robust postgraduate degree in science communication [23]. However, these programs by themselves will not necessarily address the problem I highlight above, namely, training a cadre of working scientists equipped with the needed tools to engage the public and become recognized science champions. We must shape a new ecosystem of public engagement and communication for scientists who actively conduct experimental research or head laboratories. A key element of this would include building new public communications tracks in science PhD or MD-PhD programs or creating options for postdoctoral fellows. We also must find ways to incentivize junior and midcareer scientists to participate in, or even lead, public engagement activities.

Currently, such training and career promotion mechanisms are lacking. Too often, graduate students and postdoctoral students, as well as junior or midcareer faculty, quickly learn that media activities or public engagement represent unwelcome distractions. Instead, they are given the message to remain focused exclusively on their grant applications and peer-reviewed scientific papers or, in some cases, are allowed some wriggle room for training and educational activities. Right now, there are few, if any, options to consider public outreach (such as writing op-eds or books; talking to journalists; and speaking on television, radio, or to the lay public) as essential activities. Metrics for these activities are generally not included in assessments of academic progress or promotion, and there are few incentives for scientists to speak out [24]. There are even fewer options for media training for scientists. However, if we are serious about fighting off antiscience and antiscience movements, we must build a system in which those activities are considered vital.

Adding to the complexity of embarking on public engagement in science is the simple truth that science itself is far from perfect. As a recent *Nature* editorial points out, our profession is only now confronting problems around irreproducible results, financial conflicts of interest, and other pressing issues [25]. Communicating science while being up front about these realities is not simple.

Not everyone is suited for public engagement, and it may be that only a modest percentage of young scientists would seek such opportunities even if offered or encouraged. Yet it is clear to me, from lectures, speeches, and interviews across the US and Europe that many young scientists are eager to take on this challenge. Anecdotally, their commitment to public service is at an all-time high, as confirmed by the 2017 March for Science. It is almost routine for me after a lecture to receive a small line of young scientists who tell me that they are “all in” and want to do this, but we do not have a system in place to make this easy. Too often, we scare away young and eager scientists who express a willingness to take on this activity [26].

## A new role for open-access publishers?

Since 2014, Dr. Bill Sullivan and his colleagues Drs. Krista Hoffman-Longtin and Jason Organ created #SciComm, a PLOS blog to encourage science communication [27]. In parallel, they are building an innovative science communication program at Indiana University Medical Center. The Alan Alda Center for Communicating Science at Stony Brook University, and possibly other centers, are also playing important roles on this front. I believe programs related to science communication and public engagement should be created for all major doctoral science or even medical school programs, while tracks in public engagement with next generation metrics must be established for junior and midcareer faculty. Potentially, this area of public engagement could become a new focus for open-access publishers, who make their content available without restriction. The need is great, and many young scientists want to develop these skills and commit to public service. In 2018, the *PLOS Biology* Staff Editors sounded a warning about the rising threat of antiscience [28]—this approach might represent an action item.

I had to learn about public engagement and communication the hard way—through decades of trial and error and finding a voice around neglected diseases and vaccines. I had to figure out, under duress, how to counter and manage aggression from the antivaccine movement. But it does not have to be this way. Currently, there is a vacuum enabling the proliferation of attacks against science, from vaccines to climate change. Therefore, while I am mostly focused on combating the antivaccine movement, in fact, antiscience activities are far more rampant and extend across many areas. Ultimately, we can help defeat antiscience movements by creating a cadre of top-flight scientists ready to engage and incentivized and promoted based on their successes.

## Abbreviations

AAAS: American Association for the Advancement of Science
HPV: human papillomavirus
MMR: measles-mumps-rubella
NASEM: National Academies of Science, Engineering, and Medicine
